# Editorial: Intelligent computing in farmland water conservancy for smart agriculture

**DOI:** 10.3389/fpls.2023.1236010

**Published:** 2023-07-20

**Authors:** Yirui Wu, Shaohua Wan, Shivakumara Palaiahnakote

**Affiliations:** ^1^ College of Computer and Information, Hohai University, Nanjing, China; ^2^ Shenzhen Institute for Advanced Study, University of Electronic Science and Technology of China, Shenzhen, China; ^3^ Department of Computer System and Information Technology, University of Malaya, Kuala Lumpur, Malaysia

**Keywords:** big data in smart water conservancy, data-based intelligent water conservancy management, water decision support in smart water conservancy management, automated monitoring technology in smart water conservancy, data correlation analysis in smart water conservancy

In the past few decades, the rapid development of agriculture has put forward high requirements for efficient management of water resources, so as to rationally utilize natural resources and increase their sustainability. It is noted that there is a wide gap between demand and water supply, which leads to water scarcity in agriculture. The key reason lies in the difficulty to predict variations in nature. Therefore, there is a need for smart farmland water management. A trustworthy smart farmland water conservancy model should meet the following six goals: spatial globalization, time serialization, process automation, application intelligence, management integration, and scientific decision-making. Based on these six goals, smart water conservancy should satisfy the automation, refinement, real-time, and comprehensiveness of water resources management.

This Research Topic includes four papers after peer reviewed that focus on smart farmland water management. “*A water quality assessment method based on an improved grey relational analysis and particle swarm optimization multi-classification support vector machine*” written by Gai et al. proposes a river water quality assessment method based on improved grey correlation analysis (ACGRA) andparticle swarm optimization multi-classification support vector machine (PSO-MSVM) for assessing river water environment quality. Their paper offers a proper machine learning based method to accurately evaluate the water environment quality.

Since reservoir operation is important for basin water resources management, Hu et al. writes “*A decision-making method for reservoir operation schemes based on deep learning and whale optimization algorithm*”, which proposes a reservoir operation scheme decision-making model IWGAN-IWOA-CNN based on artificial intelligence and deep learning technology. Experiments show their method has higher prediction accuracy and reliability of scheme selection.

Inspired by sequence prediction task with deep learning methods, Yuan writes “*A novel pyramid temporal causal network for weather prediction*”, which proposes Pyramid Temporal Causal Network (PTCN) to address the weather prediction issue affecting water management. Their method greatly improves the prediction accuracy with respect to small variance variables.

Accurate prediction of soil salinity and crop evapotranspiration under drip irrigation is essential to guide water management practices in arid and saline areas. Jiang et al. writes “*Simulating soil salinity dynamics, cotton yield and evapotranspiration under drip irrigation by ensemble machine learning*”. Based on a global dataset collected from 134 pieces of literature, their method comprehensively simulates soil salinity, evapotranspiration (ET) and cotton yield. The accuracy of their model has reached a satisfactory level, R2 in 0.78-0.99.

Overall, have released four excellent papers on our Research Topic, which show promising development towards smart farmland. However, there are still open challenges. Due to differences in climate, terrain, soil, and water resource conditions in different regions, intelligent computing technology for agricultural water conservancy needs to be customized and developed in conjunction with local actual conditions. Indeed, more research is needed on intelligent models in smart water conservancy or smart farmland water conservancy to realize the beautiful vision of interconnected perception and harmony between human and water. To further explore the discipline, we hope researchers and practitioners from academia and industry can carry on this Research Topic for developing. We thank authors of the papers published in the Research Topic and journal team.

## Author contributions

All authors listed have made a substantial, direct, and intellectual contribution to the work and approved it for publication.

